# Recombinant Human GH in Managing Refractory Hypoglycemia in a Young Patient With Embryonal Rhabdomyosarcoma

**DOI:** 10.1210/jcemcr/luaf137

**Published:** 2025-07-28

**Authors:** Aditya V Belamkar, Viral N Shah, Palak Patadia, Avery Abfall, Adelina Priscu

**Affiliations:** Department of Medicine, Indiana University School of Medicine, Indianapolis, IN 46202, USA; Division of Endocrinology & Metabolism, Indiana University School of Medicine, Indianapolis, IN 46202, USA; Center for Diabetes and Metabolic Diseases, Indiana University School of Medicine, Indianapolis, IN 46202, USA; Division of Endocrinology & Metabolism, Indiana University School of Medicine, Indianapolis, IN 46202, USA; Department of Health and Human Sciences, Purdue University, Indianapolis, IN 47907, USA; Division of Endocrinology & Metabolism, Indiana University School of Medicine, Indianapolis, IN 46202, USA

**Keywords:** non-islet cell tumor hypoglycemia, embryonal rhabdomyosarcoma, IGF-2, somatropin, recombinant human growth hormone

## Abstract

Non-islet cell tumor hypoglycemia (NICTH) is a rare paraneoplastic syndrome associated with various malignancies, mediated by the overproduction of IGF-2. We describe a 24-year-old male with metastatic embryonal rhabdomyosarcoma who presented with severe symptomatic hypoglycemia. Workup confirmed suppressed insulin and ketone levels, with an IGF-2/IGF-1 ratio of 3.6 (<3). Hypoglycemia was initially managed with corticosteroids and dextrose-containing fluids without success. Since surgical debulking was not feasible due to tumor burden, recombinant human GH (rhGH) was considered. Somatropin was initiated at 1 mg daily and uptitrated to 2 mg daily over 2 days, resulting in resolution of hypoglycemia with progressive tapering of dextrose-containing fluids and steroid doses. He was discharged on rhGH 2.7 mg daily with no further hypoglycemic episodes. This case highlights the challenges of NICTH, emphasizing the need for individualized treatment strategies. While the IGF-2/IGF-1 ratio did not meet the classic threshold (>10), other laboratory testing was suggestive of a non-insulin, IGF-2-mediated pathway. The use of rhGH for NICTH is not fully understood; however, it may be an important tool in preventing hypoglycemia in patients with nonresectable malignancies.

## Introduction

Non-islet cell tumor hypoglycemia (NICTH) is a rare phenomenon associated with various malignancies. This process is mediated through IGF-2, which, in normal physiology acts as a minor growth factor with a secondary role to IGF-1 [1]. IGF-1 is typically regulated by the hypothalamus-pituitary axis mediated by GH, while IGF-2 is not normally responsive to GH [2]. However, in NICTH, IGF-2 is grossly overexpressed by tumor cells, especially of mesenchymal and epithelial origin, exerting an insulin-like effect on the body, promoting hypoglycemia by binding both IGF receptors and insulin receptors. The pathophysiology of NICTH is centered on overproduction of IGF-2 with suppression of both insulin and IGF-1 [3, 4]. The interaction between IGF and insulin signaling pathways and their role in cancer progression has been previously documented [2]. This often presents as symptomatic fasting hypoglycemia, endogenous insulin and ketones suppression, and an elevated IGF-2/IGF-1 ratio. Other mechanisms of malignant hypoglycemia include wasting due to deficiency of gluconeogenic precursors and overconsumption of glucose by the tumor, often in the setting of hepatic glycogen depletion [5]. Overall, treatment of this condition is centered around reducing tumor burden, providing high carbohydrate nutrition, and medical management. These have been described with varying efficacy in the literature; however, the data is limited due to the scarcity of cases and limited long-term prognosis of many of these patients. In this case, we describe a young man with new-onset hypoglycemia in the setting of progressive, metastatic malignant disease necessitating trial of rhGH.

## Case Presentation

**Figure 1.**
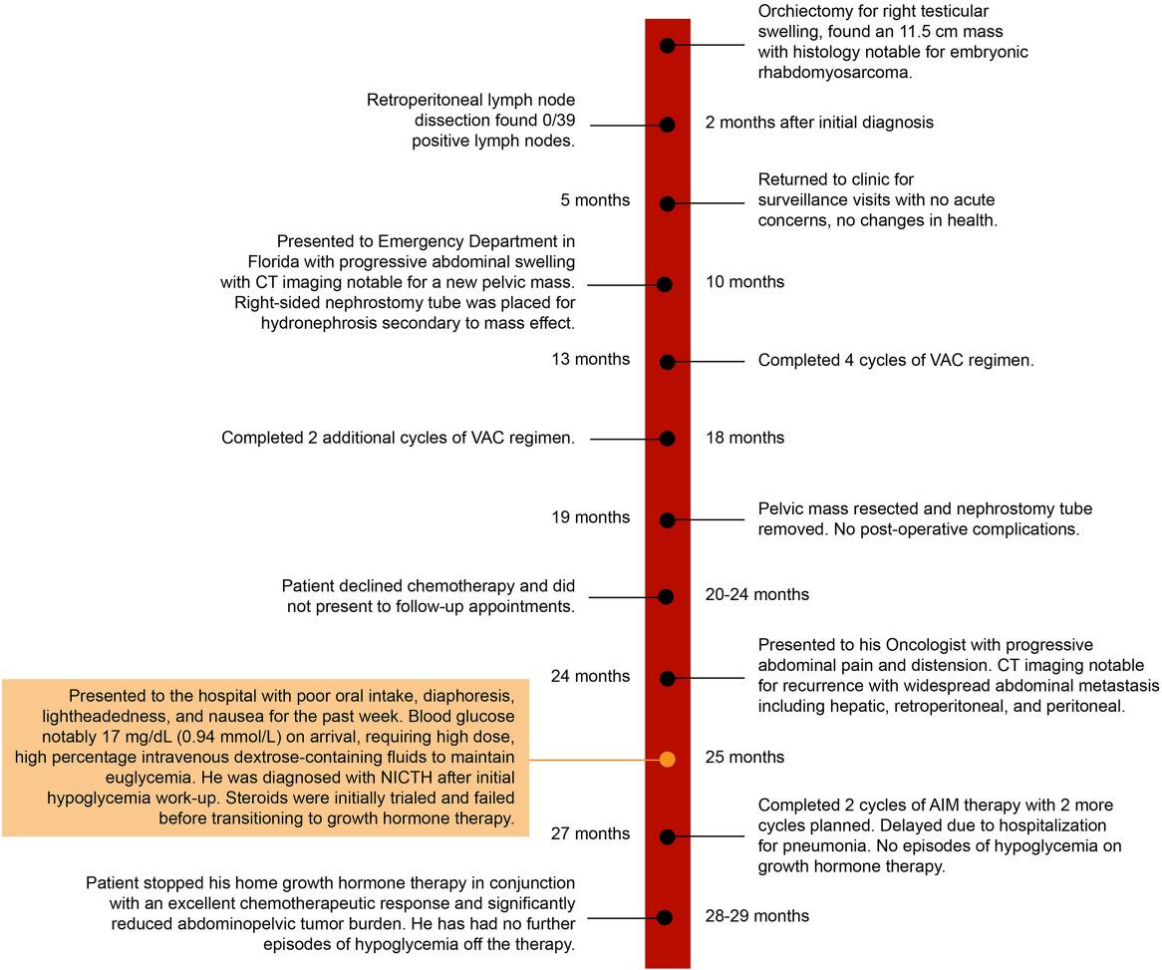
Timeline of patient's oncologic history.

A 24-year-old male with a prior diagnosis of metastatic embryonal rhabdomyosarcoma complicated by deep vein thrombosis and atrial thrombi managed with aspiration thrombectomy, apixaban, and an inferior vena cava filter and a history of gunshot wounds to the chest and abdomen presented with diffuse abdominal pain and altered mental status. His malignancy was diagnosed 3 years earlier following an orchiectomy for testicular swelling, with pathology of the 11.5 cm mass confirming a diagnosis of embryonal rhabdomyosarcoma (Fig. 1). He had 6 cycles of vincristine, dactinomycin, and cyclophosphamide, achieving initial oncologic improvement. However, his disease course was complicated by pelvic recurrence 2½ years after diagnosis and 4 months prior to his current presentation, requiring percutaneous nephrostomy tube placement, surgical resection, and retroperitoneal lymph node dissection. He subsequently declined additional chemotherapy and missed multiple follow-up appointments. Imaging 3 months postresection revealed widespread abdominal recurrence, including hepatic metastases. At this time, he was scheduled to begin additional chemotherapy, but prior to this, he presented to the hospital reporting progressive diffuse abdominal pain, 20-pound weight loss over 3 months, difficulty urinating, and altered mental status. He presented with a weight of 51.9 kg (<5th percentile for race and age) and a body mass index of 15.5 (<5th percentile), which was a significant downtrend from weights ranging in the 5th to 10th percentile in the past 6 months.

**Figure 2.**
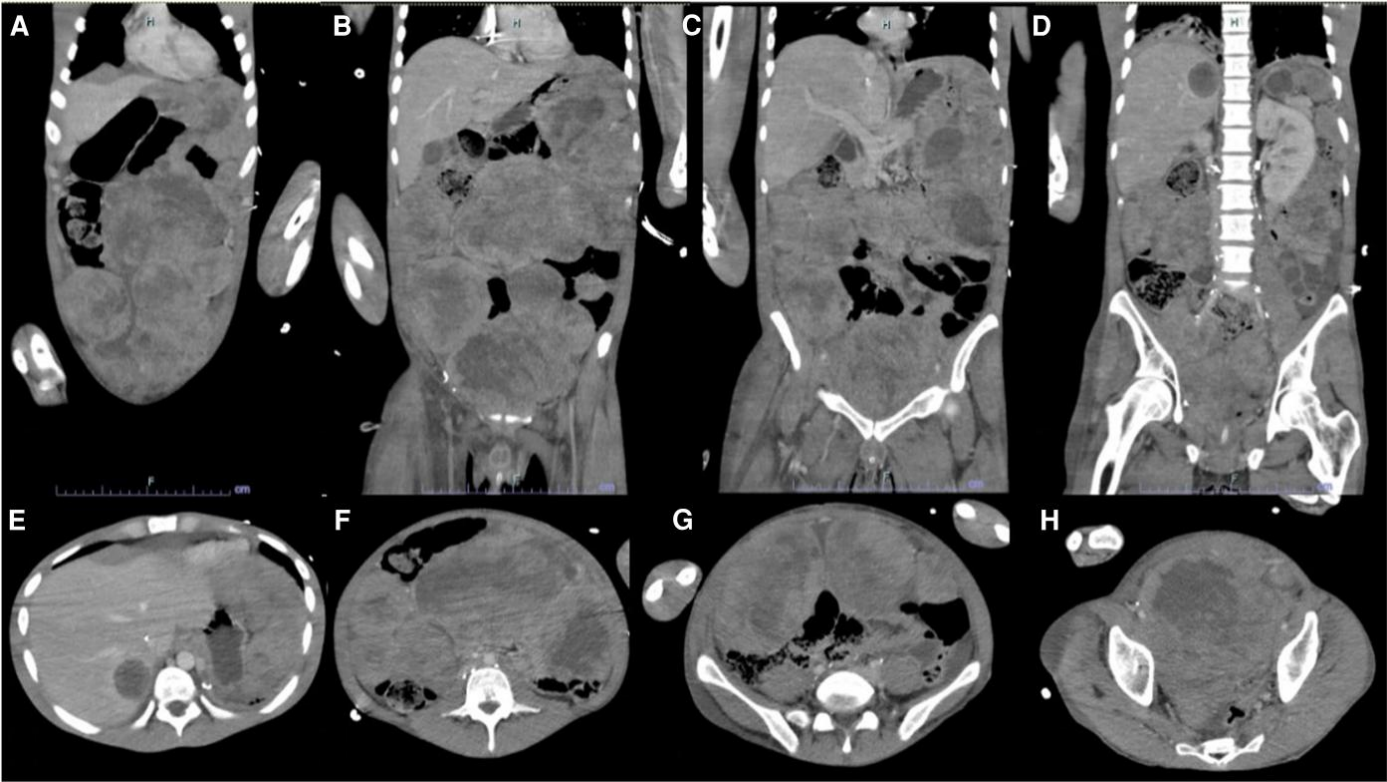
(A-H) Abdomen and pelvis computed tomography from presentation notable for significant abdominopelvic and hepatic lesions consistent with known metastatic embryonal rhabdomyosarcoma. There is also moderate right renal hydronephrosis due to mass effect that did not require decompression.

**Figure 3.**
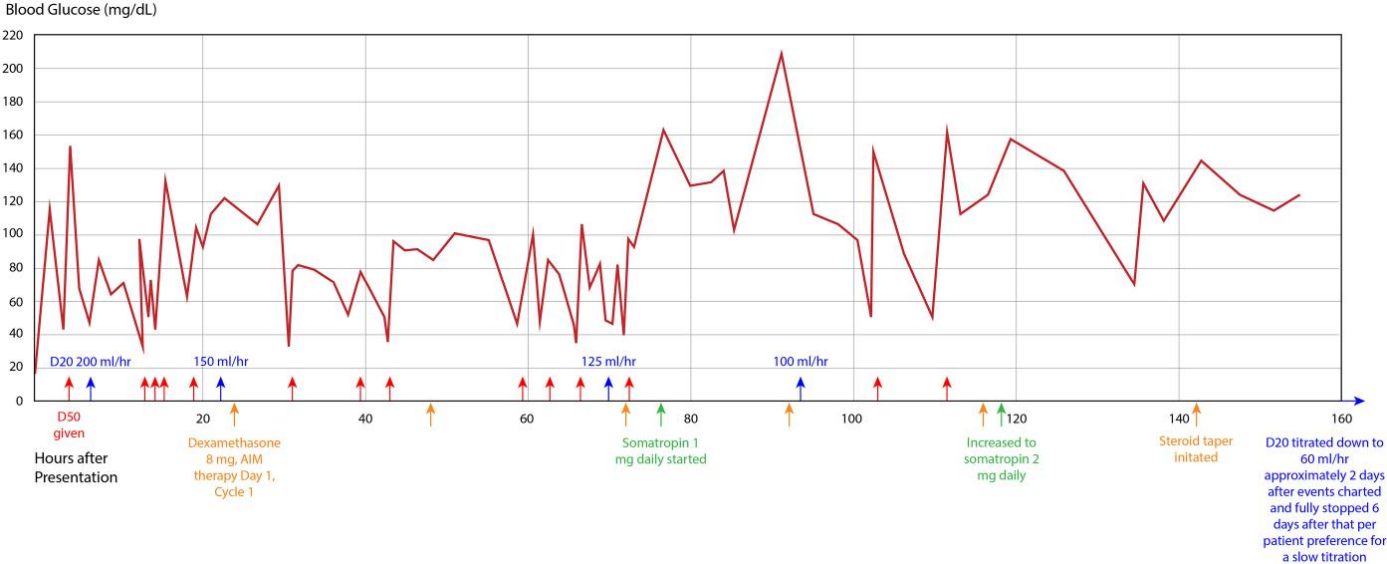
Glycemic course over the initial 160 hours following presentation including hypoglycemic events and glycemic medication and fluid treatments. Abbreviations: D20, dextrose 20%; D50, dextrose 50%; IAM, doxorubicin, ifosfamide, and mesna therapy.

On admission, the patient was hemodynamically stable but hypothermic to 34.3 °C and severely hypoglycemic (17 mg/dL (SI: 0.94 mmol/L) (reference range, 70-100 mg/dL [SI: 3.89-5.55 mmol/L]) with loss of consciousness. He had no prior history of diabetes or hypoglycemia. Abdominal and pelvic computed tomography demonstrated extensive, heterogeneously enhancing masses consistent with progressive malignant disease, moderate right-sided hydronephrosis due to mass effect, and progressive hepatic lesions consistent with prior noted metastases (Fig. 2A-2B). Persistent hypoglycemia required dextrose 50% IV pushes and dextrose 20% IV fluid at 200 mL/hour (12.85 mg/kg/min), prompting endocrinology consultation. Figure 3 describes his glycemic course including hypoglycemic management within the first 160 hours following presentation. Pertinently, he reported experiencing diaphoresis, nausea, and confusion associated with low blood glucose, which improved with glucose treatment. He endorsed social alcohol consumption and recreational marijuana use. His appetite was recently diminished due to abdominal pain and constipation, but he consumed 2 to 3 protein shakes daily along with occasional solid foods.

## Diagnostic Assessment

As the patient met criteria for Whipple triad, guideline-directed workup was initiated [6, 7]. Table 1 details notable lab work performed after pausing dextrose-containing fluids. The IGF-2/IGF-1 ratio was calculated to be 3.6, which did not align with the typical pattern of IGF-2-mediated hypoglycemia, where this ratio is expected to be greater than 10. However, it is important to note the normal ratio is 3, and IGF-1 was suppressed (Table 1) [4]. He had no evidence of renal failure even in the setting of hydronephrosis, therefore, IGF binding protein 3 level was not measured. A glucagon stimulation test was not conducted due to the significant drop in glucose.

**Table 1.** Laboratory data from in-patient hypoglycemia workup

Lab	Result	Reference range
Serum glucose	30 mg/dL (SI: 1.67 mmol/L)	70-100 mg/dL [SI: 3.89-5.55 mmol/L]
Insulin	0.50 μU/mL (SI: 3.47 pmol/L)	4.00-30.00 μU/mL [SI: 27.78-208.35 pmol/L]
Proinsulin	<2.0 pmol/L (SI: <2.0 pmol/L)	≤7.2 pmol/L [SI: ≤7.2 pmol/L]
C-peptide	0.1 ng/mL (SI: 0.03 nmol/L)	1.1-4.4 ng/mL [SI: 0.36-1.46 nmol/L]
Insulin antibody	<0.4 units/mL (SI: <0.4 units/mL)	0.0-0.4 units/mL [SI: 0.00-0.40 units/mL]
Sulfonylurea panel	Negative	
β-hydroxybutyrate	0.04 mmol/L (SI: 0.04 mmol/L)	0.02-0.27 mmol/L [SI: 0.02-0.27 mmol/L]
TSH	4.850 μU/mL (SI: 4.85 μU/mL)	0.400-4.200 μU/mL [SI: 0.40-4.20 μU/mL]
Free T4	1.1 ng/dL (SI: 14.16 pmol/L)	0.6-1.5 ng/dL [SI: 7.72-19.30 pmol/L]
Cortisol (8 **Am**)	12.3 μg/dL (SI: 339.36 nmol/L)	8-25 μg/dL [SI: 220.72-689.75 nmol/L]
Cortisol (4 **Pm**)	16.2 μg/dL (SI: 446.96 nmol/L)	4-20 μg/dL [SI: 110.36-551.80 nmol/L]
ACTH	6.7 pg/mL (SI: 1.48 pmol/L)	7.2-63.0 pg/mL [SI: 1.59-13.87 pmol/L]
IGF-1	45 ng/mL (SI: 5.90 nmol/L)	155-432 ng/mL [SI: 20.30-56.59 nmol/L]
IGF-2	162 ng/mL (SI: 21.22 nmol/L)	180-580 ng/mL [SI: 23.58-75.98 nmol/L]
IGF-2/IGF-1 ratio	3.6	<10

## Treatment

Given his extensive disease burden, debulking surgery and enteral feeding interventions were not feasible. Total parenteral nutrition was not considered due to the higher risk for infection associated with planned chemotherapy. A literature review identified potential management options, including corticosteroids, rhGH, and somatostatin analogs. The patient was already receiving IV dexamethasone 8 mg (prednisone equivalent 53.3 mg) daily per oncology chemotherapeutic protocols in addition to IV dextrose 20% fluids at rates between 150 and 200 mL/hour (9.63-12.85 mg/kg/min) without improvement in his hypoglycemia (Fig. 3). He was initiated on subcutaneous somatropin 1 mg, with subsequent titration to 2 mg daily, which allowed for slow tapering of IV dextrose fluids over 12 days and weaning off corticosteroids (Fig. 3). The patient experienced a cessation of hypoglycemic episodes with this uptitration.

## Outcome and Follow-up

He was discharged on somatropin 2.7 mg subcutaneously after a 37-day hospital course including 2 cycles of doxorubicin, ifosfamide, and mesna chemotherapy. He continued to be free of hypoglycemic episodes 3 months after starting this therapy, at which point he discontinued it in conjunction with computed tomography imaging evidence of a significant decrease in abdominopelvic tumor burden, demonstrating excellent response to chemotherapy. His weight improved to 52.3 kg (<5th percentile) at the time of discontinuation and further trended up to 56.2 kg (<5th percentile) in the subsequent months. He is scheduled for surgical resection of the remaining tumor burden in the near future. Notably, he has not had any hypoglycemic episodes since discontinuing therapy. Unfortunately, as he independently discontinued treatment and had a challenging early oncologic course, follow-up IGF levels were not obtained during therapy. Once off therapy, further testing was deemed an unjustified use of resources given his clinical status.

## Discussion

This case describes a new-onset hypoglycemia in the setting of significant abdominopelvic tumor burden with hepatic metastases managed with somatropin after failing corticosteroids and IV dextrose-containing fluids. Differential diagnosis of hypoglycemia in patients without diabetes includes insulinoma, noninsulinoma pancreatogenous hypoglycemia, postbariatric hypoglycemia, insulin autoimmune hypoglycemia, and insulin secretagogue pathways. Alternative diagnoses including infection, poor nutritional intake, and hepatic failure were considered. In this case, the diagnosis of NICTH was complicated by an IGF-2/IGF-1 ratio less than 10 but above normal limits. The overall workup correlated with the clinical picture of IGF-2-mediated hypoglycemia with suppressed insulin, C-peptide, and proinsulin with a serum glucose less than 55 (Table 1) [6, 7]. Most importantly, ketone production was suppressed, indicating activation of insulin-mediated pathways in the setting of appropriate insulin suppression, likely through IGF-2 mediation. Ketones would be expected to be elevated if this was a non-insulin, non-IGF-mediated pathway [6]. As a result, we hypothesize the laboratory assay may not appropriately detect high molecular weight IGF-2 that is most commonly expressed by tumor cells [7]. Another possibility is steroid administration prior to blood draws may have suppressed IGF-2 production [4, 8]. Finally, this may be multifactorial secondary to above normal IGF-2 production, reduced hepatic glycogen stores, and gluconeogenesis in the setting of metastases and poor nutrition, and excessive consumption from significant abdominopelvic tumor metabolic burden.

Diazoxide was not appropriate for this patient's hypoglycemia as initial laboratory data indicated suppression of endogenous insulin as well as concern that his blood pressures would not tolerate diazoxide-mediated vasodilation. Similarly, somatostatin analogs were not considered as these primarily suppress insulin secretion. Given the patient's clinical picture, oncology was in agreement with a trial of rhGH, acknowledging the risk for tumor growth being outweighed by the benefits of preventing hypoglycemia.

The consensus for managing NICTH is for initial IV dextrose management via central venous catheter, followed by surgical resection, and finally medical management [5]. Studies suggest glucagon infusion, corticosteroids, rhGH, and somatostatin analogs as potential options. A recent systematic review looking at 172 studies, primarily case reports, included 233 patients with NICTH. Surgical resection was the most common initial treatment modality, performed in 47.2% of patients [9]. Embolization and radiotherapy were both used in 6% of cases [9]. Corticosteroids were the most frequently administered first-line medical therapy (39.1%), followed by octreotide (7.7%) and diazoxide (6.9%) [9]. No patients in the study received rhGH [9]. Importantly, the study noted only those patients who received surgical intervention had significantly improved odds of recovering, while those who received corticosteroids or octreotide were significantly less likely to improve [9]. Presumably the patients who did not undergo surgical interventions were more complex and poor surgical candidates. Thus, medical management of NICTH may be seen as a palliative measure. Overall, the consensus of the literature is that surgical resection, when possible, is the most effective treatment, followed by other tumor burden reduction modalities and finally medical management [10]. The Endocrine Society Clinical Practice Guidelines do not describe the management of hypoglycemia in patients without diabetes [6].

The use of rhGH has been reported in literature despite concerns regarding its potential to promote tumor growth (Table 2) [7]. The exact mechanism by which it prevents hypoglycemia remains unclear. However, it is hypothesized that at supraphysiologic doses, rhGH suppresses peripheral glucose uptake and increases IGF-1 levels, which may help normalize IGF-2 binding dynamics by promoting the formation of binary and ternary complexes [4]. Additionally, rhGH may enhance hepatic glucose production through stimulation of glycogenolysis, gluconeogenesis, and lipolysis. The literature also suggests that glucocorticoids are effective in this context by lowering IGF-2 levels and increasing IGF-1 concentrations [4].

**Table 2.** Summary of published studies utilizing GH therapy for NICTH

*Study*	*Age and* sex	*Malignancy*	*Presentation*	*Therapy*	*Effect*	*Outcome*
*Silveira et al, 2002 [11]*	83 yo F	Retroperitoneal fibrous tumor	Hypoglycemic coma, 3-month history of symptomatic hypoglycemia	Human GH 4 units in the morning, 6 units in the evening	Improved glucose, insulin, C-peptide, IGF-1; decreased IGF-2 after 2 months of therapy	Patient passed during debulking surgery
*Agus et al, 1995 [12]*	2 yo F	Neuroblastoma	Hypoglycemic seizures	GH 0.05 mg/kg titrated up to 0.2 mg/kg divided into 2 doses	Improved glucose, IGF-1, IGFBP-3; decreased IV dextrose requirement, IGF-2	Changes maintained after 6 months of GH therapy
*Bourcigaux et al, 2005 [13]*	67 yo F	Pleural fibroma	Hypoglycemia requiring glucose infusion and regular oral carbohydrate intake	3 phases: maintenance therapy of prednisone 10 mg, and rhGH 1.3 mg daily	Improved glucose, insulin, C-peptide, GH, IGF-1, IGFBP-3	Patient had no hypoglycemic episodes 6 months after initiating treatment at which she passed from respiratory failure related to NICTH
*Hirai et al, 2016 [14]*	61 yo F	Gastrointestinal stromal tumor	Refractory symptomatic hypoglycemia for 4 months	GH 1 mg/day	Resolved hypoglycemia	Long-term outcomes not reported
*Perros et al, 1996 [15]*	74 yo F	Inconclusive histology, suspect solitary fibrous tumor of the pleura	Neuroglycopenia	Subcutaneous rhGH 2 units nightly, oral prednisolone 30 mg daily, bendrofluazide 5 mg daily	Octreotide failed initially in controlling hypoglycemia, but switching to prednisolone abolished these events; notable reduction in IGF-2; restoration of insulin secretion	Prednisolone dose was reduced to 15 mg daily without further episodes of hypoglycemia; patient lived independently for 9 months at home before passing suddenly of unknown causes
*Teale and Marks, 1998 [16]*	80 yo F	Medullary thyroid cancer	Severe, protracted fasting hypoglycemia	hGH: 12 units days 1 and 2, 24 units day 3, 36 units day 4	hGH therapy increased glucose; no effect on insulin production, increased IGF-1; variable effect on IGF-2; increased IGFBP-3	
	83 yo F	Pleural fibroma		hGH: 4 units daily for 4 days, 8 units daily for 11 days, 12 units daily for 3 days		
	64 yo M	Hepatoma		hGH: 6 units daily for 27 days		
	69 yo F	Lung carcinoma		hGH: 4 units daily for 3 days, 10 units daily for 2 days, 12 units daily for 2 days, 24 units daily for 7 days		
	30 yo M	Hemangiopericytoma		Dexamethasone: 4 mg thrice daily for 3 months, 4 mg twice daily for 4 months	Glucocorticoid therapy increased glucose, insulin production, IGF-1, IGFBP-3 and decreased IGF-2	
	92 yo F	Pleural fibroma		Prednisolone: 30 mg daily for 6 weeks		
	74 yo F	Pleural fibroma		Prednisolone: 30 mg daily for 4 months		
	81 yo F	Lung carcinoma		Prednisolone: 30 mg daily for 4 months		
*Lee and Twigg, 2015 [17]*	74 yo M	Hemangiopericytoma	Neuroglycopenic symptoms of confusion and lightheadedness	Initially trialed dexamethasone at 1 mg orally twice daily and uptitrated to 2 mg twice daily, which had limited efficacy; weaned dexamethasone to 1 mg twice daily and added rhGH 0.45 units daily	Resolution of hypoglycemia, increase in IGF-1 and IGFBP-3, and reduction of IGF-2	Improved quality of life for many months without hypoglycemia
*Powter et al, 2013 [18]*	27 yo F	Ovarian yolk sac tumor	Symptomatic hypoglycemia, passed out	Trialed dexamethasone at 4 mg twice daily and uptitrated to 16 mg twice daily to prevent recurrence of hypoglycemia; however, due to recurrence of symptoms, rhGH 0.4 units/kg/day was initiated allowing dexamethasone to be titrated down	Maintained normoglycemia	Therapy allowed patient to travel to her home country where she passed away with her family after a few days
*Mukherjee et al, 2005 [19]*	75 yo M	Leydig cell tumor	Found unconscious, severely hypoglycemic	Initially treated with IV D10 and dexamethasone 2 mg 4 times daily but was unable to come off the IV D10; he was started on rhGH 8 units that increased to 32 units daily, allowing for the dextrose infusion to be stopped gradually	Had no more hypoglycemic episodes	Opted for conservative approach; passed away 10 days after admission due to deteriorating renal function
*Berman and Harland, 2001 [20]*	46 yo F	Renal cell carcinoma	Confusion and expressive dysphagia, hypoglycemic	Started on diazoxide 100 mg thrice daily with continued symptomatic nocturnal hypoglycemia; added cornstarch at night without improvement; subsequently prednisolone 20 mg was added with symptomatic improvement; however, this lasted only 10 days before another episode of severe symptomatic hypoglycemia, prompting the addition of bendrofluazide 5 mg daily and GH 2 units nightly	Symptoms did not improve on GH and patient was switched to octreotide 100 units nightly and increased prednisolone to 40 mg daily	Patient continued to have symptomatic hypoglycemia and trialed a variety of carbohydrate administration; after 1 month her condition deteriorated and patient passed away
*Hunter et al, 1994 [21]*	64 yo M	Hepatoma	Unconscious; found to be hypoglycemic	Initially trialed high-calorie diet, followed by GH 2 units daily, GH was uptitrated to 2 units thrice daily	Even with uptitration, octreotide 50 micrograms was added without benefit, leading to administration of IV dextrose for discharge	Remained free from hypoglycemia for 24 months after his initial presentation before subsequently passing away
*Drake et al, 1998 [22]*	65 yo M	Benign pleural tumor	Acute confusional state resulting from hypoglycemia	rhGH 6 units daily	Increased mean plasma glucose, IGF-1, IGFBP-3	Attempted debulking surgery; patient developed pneumonia and died on postoperative day 12
	54 yo M	Pleural fibrosarcoma	Not arousable and sweating due to hypoglycemia	Hypoglycemia worsened by octreotide 100 mcg; rhGH 8 units daily successfully alleviated hypoglycemia	Increased mean plasma glucose, IGF-1, and IGFBP-3	Prevented hypoglycemia for 5 months after initially starting rhGH before development of respiratory failure and recurrent hypoglycemia requiring 12 units daily; passed away at this time due to respiratory failure
*Katz et al, 1996 [23]*	2 yo F	Neuroblastoma	Hypoglycemic seizure	GH 0.2 mg/kg/day twice daily	Alleviated hypoglycemia; able to fast 12 hours without hypoglycemia	Discharged and after 5 months decreased GH to 0.1 mg/kg/day twice daily; maintained on this treatment for 1 year without side effects and improved growth
*Dilrukshi et al, 2020 [24]*	43 yo M	Adrenocortical carcinoma	Severe nonketotic, noninsulin-mediated hypoglycemia, also ACTH-dependent Cushing's syndrome	rhGH 3 mg/daily, increased to 4 mg/daily	Significant reduction of severity and frequency of hypoglycemia	Opted for palliative chemotherapy; however, passed away due to illness while receiving this treatment
*Wong, 2015 [25]*	56 yo F	Pleural mesothelioma	Confusion, diaphoresis, weakness	Trialed prednisone 30 mg uptitrated to 100 mg daily without success as well as adding diazoxide; successful treatment occurred with dexamethasone 16 mg/day and rhGH 2.65 mg/day	Stabilization of average blood glucose level with range within normal limits	Died due to complications related to mesothelioma week after starting final therapy; no further episodes of hypoglycemia
*Vu et al, 2024 [8]*	28 yo F	Solitary fibrous tumor	Unresponsive with severe hypoglycemia	Complete tumor resection and retroperitoneal lymph node dissection	Symptoms resolved after resection	Euglycemic with no evidence of tumor recurrence 1 year after surgery
	54 yo F	Hemangiopericytoma	2-week history of intermittent confusion, decreased responsiveness, and speech difficulty occurring overnight while fasting	Steroids resulted in psychiatric adverse effects, no improvement with octreotide 100 mcg, switched to rhGH 0.67 mg daily and titrated up to 1.67 mg daily in combination with dexamethasone 1 mg daily	Prevented nocturnal hypoglycemia and allowed for dextrose to be discontinued	Hypoglycemia was prevented with rhGH and low-dose dexamethasone; passed away 6 months later under palliative care
*Dimitriadis et al, 2015 [26]*	70 yo F	Gastrointestinal stromal tumor	Left-sided vasomotor symptoms including reduced muscle tone, weakness, and slurring of speech, progressive over past 3 months	Diazoxide failed initially, prednisolone 20 mg daily was successful in controlling symptoms and weaning IV dextrose; however, to minimize steroid side effects, rhGH was added with tapering of prednisolone dosing	Prevented hypoglycemic episodes; symptoms resolved	Imatinib significantly improved symptoms as well as tumor burden and allowed for rhGH to be discontinued; patient is doing well 8 years following diagnosis

Abbreviations: D10, dextrose 10%; F, female; hGH, human GH; IGFBP-3, IGF binding protein-3; M, male; NICTH, non-islet cell tumor hypoglycemia; rhGH, recombinant human GH; yo, years old.

Embryonal rhabdomyosarcoma is a rare cause of NICTH with only 1 other case being noted in a pediatric patient [27]. More common causes are fibrous tumors classically associated with Doege-Potter syndrome (53.2%), followed by nonfibrous tumors of hepatic origin (9%), hemangiopericytomas (8.5%), and mesotheliomas (4.7%) [9]. Overall, this is a rare paraneoplastic syndrome that can occur in a wide variety of malignancies.

In summary, this case report discusses a 24-year-old male with metastatic embryonal rhabdomyosarcoma that presented with new-onset symptomatic hypoglycemia requiring significant IV dextrose support. After failing corticosteroids per oncologic protocols, somatropin was started at 1 mg, then uptitrated to 2 mg, to wean the patient off IV dextrose. He was discharged on somatropin 2.7 mg daily and maintained euglycemia for 3 months in association with improvement in tumor burden with chemotherapy. The use of rhGH appears to be an effective therapy for preventing hypoglycemia in patients with high suspicion for NICTH who do not respond to other conventional therapies. While there is a theoretical possibility of tumor growth, in this limited case report, it does not appear that this risk affects the potential for chemotherapeutic reduction in tumor burden.

## Learning Points

- NICTH is a rare paraneoplastic syndrome classically associated with fibrous tumors but may be associated with a whole range of malignancies.
- Although NICTH is typically diagnosed with an IGF-2/IGF-1 ratio greater than 10, a ratio below this threshold does not exclude the diagnosis, particularly in the presence of fasting hypoglycemia with suppressed insulin and ketones, findings consistent with IGF-2 mediated effects.
- Somatropin represents an effective alternative to dextrose-containing fluids and corticosteroids for the prevention of hypoglycemia. However, it is important to note that patients may still respond favorably to chemotherapy, despite concerns regarding potential tumor-promoting effects.

## Data Availability

Original data generated and analyzed during this study are included in this published article.
